# Transnasal Humidified Rapid-Insufflation Ventilatory Exchange Versus Conventional Facemask Breathing for Preoxygenation During Rapid Sequence Induction

**DOI:** 10.7759/cureus.43063

**Published:** 2023-08-07

**Authors:** Diksha Karlupia, Kamakshi Garg, Richa Jain, Anju Grewal

**Affiliations:** 1 Anaesthesiology, Dayanand Medical College and Hospital, Ludhiana, IND; 2 Anaesthesiology, All India Institute of Medical Sciences, Bathinda, Bathinda, IND

**Keywords:** spo2, emergency laparotomy, pao2, thrive, preoxygenation, pulse oximetry, blood gas analysis, nasal cannula, face mask, rapid sequence induction

## Abstract

Introduction: Transnasal humidified rapid-insufflation ventilatory exchange (THRIVE), if used for pre-oxygenation and apnoeic oxygenation, has the propensity to extend the safe apnoea time and thereby decrease the incidence of desaturation during rapid sequence induction (RSI) for emergency surgeries. Hence, we proposed to evaluate the comparative efficacy of pre-oxygenation with the use of conventional facemask breathing versus THRIVE during RSI in patients undergoing general anaesthesia (GA) for emergency surgeries.

Materials and methods: Eighty patients undergoing RSI under GA for emergency abdominopelvic surgery were divided randomly into two groups. Patients were preoxygenated for three minutes with 100% oxygen via either a high-flow nasal cannula at a flow of 60 L/minute using THRIVE or a tightly-held, snuggly-fitting facemask at a flow of 12L/minute using a circle system. RSI was administered followed by laryngoscopy and endotracheal intubation. Arterial partial pressure of oxygen (PaO2) measured immediately after successful endotracheal intubation was our primary outcome. The lowest peripheral oxygen saturation (SpO2), apnoea time, number of attempts at laryngoscopy, use of any rescue manoeuvres, and any adverse event were also recorded. Data thus collected were statistically analysed.

Results: No statistically significant difference in PaO2 value was observed after successful intubation, lowest SpO_2_, apnoea time, number of attempts at laryngoscopy, use of any rescue manoeuvres, and adverse event between both the groups (p>0.05).

Conclusion: We conclude that though not superior to conventional facemasks, THRIVE is a safe, practicable, and efficient pre-oxygenation tool during RSI of GA for patients undergoing emergency surgeries.

## Introduction

Rapid sequence induction (RSI), routinely performed for patients undergoing emergency surgeries, can be a challenging situation for an anaesthesiologist [1,2]. Physiological and pathological derangements in these patients predispose them towards a higher risk of developing hypoxia during RSI, thereby limiting the safety margins during attempts to secure a definitive airway. Therefore, preoxygenation is mandatory as it prolongs the safe apnoea period and reduces intubation-associated oxyhaemoglobin desaturation [3].

Currently, preoxygenation is routinely advocated with conventional facemasks using oxygen at flow rates up to 15 L/minute. However, this flow may not be adequate for maintaining oxygenation during the RSI of these critically ill patients [4].

Transnasal humidified rapid insufflation ventilatory exchange (THRIVE) is emerging as an alternative method for preoxygenation and apnoeic oxygenation during general anaesthesia [5]. A review of the literature revealed few studies comparing conventional facemasks with THRIVE as a method for preoxygenation during RSI in terms of arterial partial pressure of oxygen (PaO2), arterial partial pressure of carbon dioxide (PaCO2), lowest peripheral oxygen saturation (SpO2) and oxygen desaturation [6-8]. Though current studies are very encouraging and promise a clinical benefit on patient outcomes, randomized trials are still needed to demonstrate that THRIVE provides a better way of preoxygenation in different population sets.

Hence, we conducted a randomized comparative study to evaluate the efficacy of preoxygenation with conventional facemask ventilation compared to THRIVE during RSI in patients undergoing general anaesthesia for emergency surgeries.

## Materials and methods

This randomized controlled trial was registered in the Clinical Trials Registry, India (registration number: CTRI/2021/09/036620), after being approved by the Institutional Ethics Committee of Baba Farid University of Health Sciences, Faridkot, Punjab, India (approval number: BFUHS/2K21p-TH/201). The procedures followed were in accordance with the Helsinki Declaration of 2003. Eighty adult patients requiring RSI for emergency abdominopelvic surgeries between September 2021 and August 2022 were included in the trial. Patients who were pregnant, with severe cardiorespiratory disease, laryngotracheal stenosis, clinical suspicion of or confirmed diagnosis of base of skull fracture or severe facial trauma that precludes nasal cannula placement, with laryngotracheal stenosis or benign or malignant hypopharyngeal obstruction, and BMI ≥ 40 kg/m^2^ were excluded from the trial.

**Figure 1:**
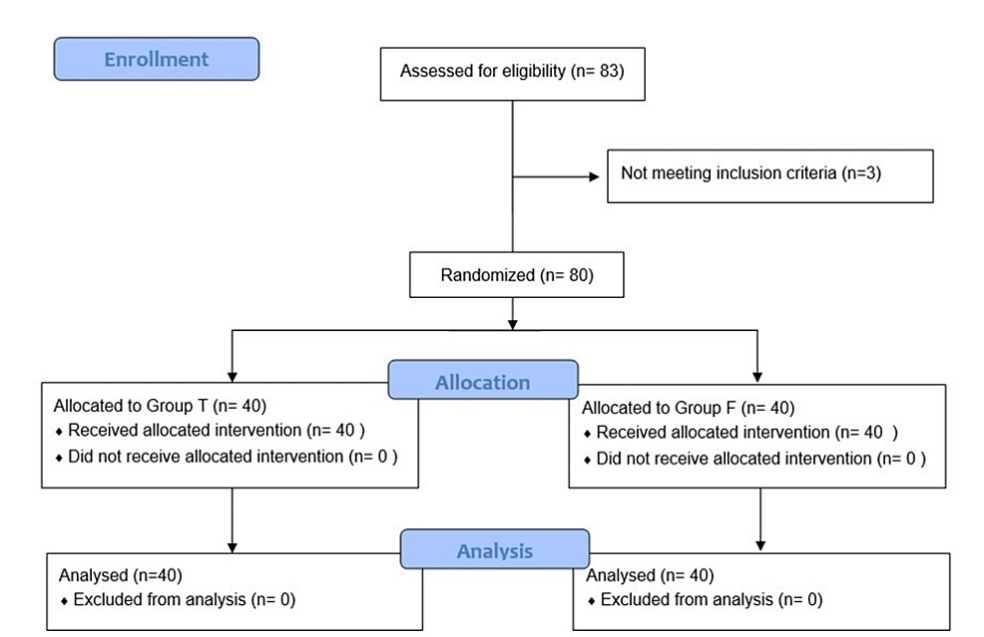
CONSORT flow diagram of the study CONSORT: Consolidated Standards of Reporting Trials

Patients were randomly allocated into one of the following two groups: (i) Group T (THRIVE) and Group F (Facemask) by computer-generated random numbers contained in sealed, sequentially numbered envelopes. The sample size calculation was based on the difference in the PaO2 (primary outcome of our trial) after preoxygenation with THRIVE and facemask. Assuming α‑error (significance) of 0.05, power (1‑ ß) of 0.95, and taking the mean difference of 86 mmHg at a 95% confidence interval as reported in a previously published study [9], the sample size required was 34 in each group. To allow for a predicted dropout from the trial, we calculated the required sample size to be 40 patients in each group. A total of 83 patients were enrolled for the study and three out of 83 were excluded as they did not meet the inclusion criteria. Finally, 80 patients were included, with 40 in each group (Figure 1).

In the operating room, the American Society of Anaesthesiologists (ASA) standard monitoring in the form of continuous five-lead ECG, non-invasive blood pressure (NIBP), and pulse oximetry was instituted. An arterial catheter was then placed in the radial artery under local infiltration anaesthesia and an arterial blood gas (ABG) sample was sent. According to the allotted group, patients were preoxygenated for three minutes with 100% oxygen via either a high-flow nasal cannula at a flow of 60 L/minute using THRIVE or a tightly-held, snuggly-fitting facemask at a flow of 12 L/minute using a circle system.

Patients in both groups were maintained on jaw thrust to ensure airway patency throughout the period of apnea. If the nasogastric tube was in situ, it was attached to continuous low-grade suction to decompress the stomach. RSI was accomplished using intravenous (IV) etomidate (0.2-0.4 mg/kg) or propofol (1.5-3.5 mg/kg) and fentanyl (2 µg/kg) followed by administration of neuromuscular blocker (rocuronium 1 mg/kg or succinylcholine 1.5 mg/kg as per patient profile).

In Group T, oxygen flow was kept at 60 L/minute during the apnoeic period and laryngoscopy whereas in Group F, patients continued to receive oxygen flow at 12 L/minute through facemask during apnoea, and bag-mask ventilation was not performed. Laryngoscopy and tracheal intubation was performed one minute after the administration of the neuromuscular blocker by an experienced anaesthesiologist. After confirming successful tracheal intubation by well-formed four capnography waveforms on the monitor, an ABG sample from the arterial catheter in situ was sent. Continuous monitoring of heart rate (HR), blood pressure, end-tidal carbon dioxide (ETCO2), and SpO2 with waveform tracing were recorded at one-minute intervals at all times after recording baseline value, until five minutes after intubation.

A drop in SpO2 to ≤ 90% during the study period was recorded as the end of apnoea and gentle intermittent positive pressure ventilation limiting the inflation pressure to 15 cmH2O using bag-mask ventilation was instituted as a rescue. If bag-mask ventilation was ineffective, an appropriate-sized second-generation laryngeal mask airway (LMA) was inserted to rescue desaturation. Laryngoscopy and endotracheal intubation was commenced after SpO2 was ≥ 98% eventually.

PaO2 measured from the ABG sample obtained immediately after successful endotracheal intubation was the primary outcome. Other observations recorded were as follows: (a) the lowest SpO2 measured until five minutes after endotracheal intubation, (b) apnoea time defined as the period from the start of apnoea till the appearance of well-formed waveform capnograph immediately after tracheal intubation, (c) the number of attempts at laryngoscopy, each attempt defined as the passage of laryngoscope through the mouth, (d) use of any rescue manoeuvres including bag-mask ventilation or use of second-generation laryngeal mask airway for ventilation, (e) adverse events like a drop in SpO2 (≤ 90%), haemodynamic instability like a drop in mean arterial pressure (MAP) <20% from baseline, and use of vasopressors, and (f) Portsmouth-Physiological and Operative Severity Score for the enumeration of Mortality and morbidity (P-POSSUM) calculated using an online calculator.

Statistical analysis

Categorical data were presented as frequency (N) and percentage (%). Continuous data were presented as mean with standard deviation (SD) or median with interquartile range (IQR). The Kolmogorov-Smirnov test was used to determine whether the data were normally distributed. Student t-test and Mann-Whitney U test were used to compare continuous variables between the study groups for independent samples for non-skewed and skewed data, respectively. Student t-test was performed to compare age while other demographic variables, ABG parameters, lowest SpO2, apnoea time, attempts of laryngoscopy and P-POSSUM were compared using the Mann-Whitney U test. For comparing categorical data such as gender, ASA grade, airway assessment parameters (modified Mallampati score and neck circumference), rescue manoeuvre, and adverse events (SpO2 < 90% and use of vasopressors). Chi-square (χ2) test was performed and Fisher's exact test was used when the expected frequency was less than 5. P-value <0.05 was considered statistically significant. All statistical calculations were done using IBM SPSS Statistics for Windows, Version 21.0 (Released 2012; IBM Corp., Armonk, New York, United States).

## Results

The characteristics of the included patients are given in Table 1.

**Table 1:** Patient characteristics in the study groups ASA: American Society of Anaesthesiologists; PaO2: arterial partial pressure of oxygen; PaCO2: arterial partial pressure of carbon dioxide; HCO3: bicarbonate

	Group F (N =40)	Group T (N=40)	P-value
Age (years), mean (SD)	50 (15.68)	51.85 (16.23)	0.61
Gender, N (%)			0.498
Female	18 (45)	15 (37.5)	
Male	22 (55	25 (56.5)	
ASA grade, N (%)			0.165
II	5 (12.5)	2 (5)	
III	19 (47.5)	27 (67.5)	
I V	16 (40)	11 (27.5)	
BMI (kg/m^2^), mean (SD)	25.955 (5.41)	25.53 (4.89)	0.713
Modified Mallampati score, N (%)			0.132
I	3 (7.5)	1(2.5)	
II	12 (30)	19 (47.5)	
III	16 (40)	17 (42.5)	
IV	9 (22.5)	3 (7.5)	
Neck circumference, N (%)			0.556
>40 cm	1 (2.5)	2 (5)	
<40 cm	39 (97.5)	38 (95)	
Cormack Lehane score, N (%)			0.077
I	7 (17.5)	3 (7.5)	
II	27 (67.5)	23 (57.5)	
III	6 (15)	14 (35)	
Baseline PaO2 (mmHg), mean (SD)	101.9 (48.77)	86.23 (27.44)	0.080
Baseline PaCO2 (mmHg), mean (SD)	34.9(5.96)	32.83 (5.02)	0.096
Baseline pH, Mean (SD)	7.435 (0.08)	7.46 (0.07)	0.111
Baseline HCO3 (mmol/L), mean (SD)	23.725 (4.34)	24.05 (5.05)	0.762
Baseline lactate (mmol/L), mean (SD)	1.2625 (1.12)	1.44 (0.86)	0.430

Mean PaO2 (primary outcome) was 239±107.07 mmHg in Group F and 258±105.77 mmHg in Group T. Thus, there was no statistically significant difference in mean PaO2 between the groups (P>0.05). PaCO2, pH, bicarbonate (HCO3), and lactates in Group F were 44.75±6.50 mmHg, 7.34±0.08, 24.73±3.92 mmol/L, and 1.20±0.80 mmol/L, respectively, whereas in Group T, they were 43.81±10.35 mmHg, 7.35±0.08, 25.22±4.71 mmol/L, and 1.42±0.91 mmol/L, respectively, which was statistically insignificant. The lowest median SpO2 was found to be 97.5% in the facemask group and 97% in the THRIVE group, which was statistically insignificant (Table 2).

**Table 2:** Outcomes of the study groups PaO2: arterial partial pressure of oxygen; PaCO2: arterial partial pressure of carbon dioxide; HCO3: bicarbonate; IQR: interquartile range; P-POSSUM: Portsmouth-Physiological and Operative Severity Score for the enumeration of Mortality and morbidity; Spo2: peripheral oxygen saturation; LMA: laryngeal mask airway

	Group F (N =40)	Group T (N=40)	P-value
After successful intubation, PaO_2 _(mmHg), mean (SD)	239.35 (107.7)	258.08 (105.77)	0.434
After successful intubation, PaCO_2 _(mmHg), mean (SD)	44.75 (6.50)	43.81 (10.35)	0.627
After successful intubation, pH, mean (SD)	7.34 (0.08)	7.35 (0.08)	0.817
After successful intubation, HCO_3_ (mmol/L), mean (SD)	24.73 (3.92)	25.22 (4.71)	0.616
After successful intubation, lactates (mmol/L), mean (SD)	1.20 (0.80)	1.42 (0.91)	0.249
Lowest Spo2 (%), median (IQR)	97.5 (93.25-99)	97 (94-99)	0.485
Apnoea time (seconds), median (IQR)	88.5 (74.25-113.75)	100 (80-120)	0.166
Number of attempts at laryngoscopy, median (IQR)	1 (1-1)	1 (1-1)	0.728
Use of any rescue manoeuvres			0.314
LMA (yes/no) (N)	No (40)	No (40)	
Bag mask (yes/no) (N)	No (39)	Yes ( 1)	
P-POSSUM score predicted mortality ratio, mean (SD)	27.31 (17.71)	27.54 (14.15)	0.950
P-POSSUM score predicted morbidity ratio, mean (SD)	74.01 (15.83)	76.31 (13.84)	0.490

## Discussion

Our study included patients undergoing RSI for emergency abdominopelvic surgeries as induction of GA in these patients is challenging due to their suboptimal circumstances and potential physiological derangements caused by their underlying illness [6]. A critically ill patient requiring emergency surgery is always considered as "full stomach"; therefore, positive pressure ventilation is usually prohibited and RSI is recommended in these sets of patients [10]. Oxygen saturation can rapidly drop to a critical hypoxic value of less than 70% within seconds in these patients [11]. THRIVE is receiving growing attention as an alternative respiratory support in critical care settings and is used in patients with different underlying diseases [12]. There are several studies in the literature confirming THRIVE as a better and more comfortable oxygenation device with lesser adverse effects [13-16]. Most of the benefits of THRIVE are attributed to positive airway pressure (< 3 cmH2O) generated by high flow delivered through a high-flow nasal cannula. This application of positive airway pressure may cause recruitment of atelectatic areas and limit end-expiratory alveolar closure [17-20].

Preoxygenation is a pivotal component of RSI in order to increase the oxygen reservoir in the lungs for extending the safe apnoea time [1]. In our study, patients in the facemask group were preoxygenated at flow rates of 12 L/minute with 100% oxygen whereas in the THRIVE group, flow was kept at 60 L/minute from preoxygenation and throughout the apnoea period. A study conducted by Ng et al. showed a significant rise in carbon dioxide (3.06 mmHg/minute) during the apnoeic period in THRIVE group when oxygen flows were kept at 50L/minute [21]. On the other hand, less increase in carbon dioxide (1.13 mmHg/minute) was seen with oxygen flows at 70L/minute in a study done by Patel et al. [22]. So we kept flows of THRIVE at 60L/minute throughout the study period as lower flow rates could theoretically affect the carbon dioxide washout [22-24].

In our study, PaO2 (as obtained from the ABG analysis) and SpO2 after successful intubation were compared as indicators of the efficacy of preoxygenation in both groups. We observed no significant difference in PaO2 and SpO2 values between both the groups and our findings were in concordance with results shown by previous studies [6-9,25,26].

Similar to our study, Mir et al. [6] and Lyon et al. [25] evaluated PaO2 as a marker of efficacy of preoxygenation with THRIVE in patients undergoing GA for emergency and non-emergency surgeries, respectively. Mir et al. compared PaO2, PaCO2, pH, and lactates post intubation between both groups and found no significant difference between them [6]. In the study by Lyon et al., subjects were divided into three groups: facemask, high-flow nasal oxygenation, and high-flow nasal oxygenation with mouthpiece [25]. They compared PaO2 values after three minutes of preoxygenation among the three groups. Hua et al. also explored the efficacy of preoxygenation with conventional facemasks and THRIVE during GA in elderly patients (65-80 years) [9]. All the aforementioned authors observed no significant change in PaO2 with THRIVE when compared with facemask. Moreover, in contrast to our study Yasser et al. studied THRIVE in pregnant patients undergoing cesarian section under GA [27]. They observed significantly increased PaO2 (p<0.01) after three minutes of apnoea period when compared with facemask with no difference in PaCO2. The reason for this might be that THRIVE could provide a stable high fraction of inspired oxygen (FiO2), positive end-expiratory pressure effect, washout of anatomical dead space, and bypass of a suboptimal seal when compared to the standard facemask preoxygenation technique in pregnant females with lower baseline functional residual capacity.

Other studies have mainly observed the early drop of peripheral saturation during apnoea as their primary outcome in both groups [7,25,26]. In line with our findings, Lodenius et al. observed a statistically insignificant difference in the median lowest SpO2 during apnoea until one minute after the tracheal intubation following preoxygenation with traditional facemask and THRIVE in patients undergoing RSI for emergency surgeries [7]. Our finding was also consistent with a comparative study by Sjoblom et al. who observed a non-significant difference in the lowest median SpO2 values between conventional facemask and THRIVE preoxygenation on 350 adult patients undergoing RSI for emergency surgeries [8].

Patel et al. [21] applied THRIVE for preoxygenation in 25 adult patients with difficult airway anatomy and found THRIVE to safely extend apnoea time up to 17 minutes without any patient desaturating ≤90%. Rajan et al. also reported a significant increase in safe apnoea with THRIVE when compared with conventional facemasks along with nasopharyngeal catheters [28]. However, we did not study the safe apnoea period in our critically ill patients and the apnoea time noted was recorded. We did not find a significant difference between apnoea time in both groups. Contrary to our study, Mir et al. observed a significant increase in apnoea time in THRIVE group and explained it was due to knowledge bias resulting in controlled and careful laryngoscopy in the THRIVE group [6].

The number of attempts of laryngoscopy and the use of manoeuvres to rescue desaturation below 90% were comparable in both groups. The trachea of all patients were intubated at the first attempt of laryngoscopy in our study. These findings were similar to observations reported by Lodenius et al. [7], Hua et al. [9], and Sjöblom et al. [8]. Though one of the obese patients in the THRIVE group in our study desaturated below 90% and bag-mask ventilation was used as a rescue manoeuvre, this difference was insignificant in both groups. In contrast, none of the patients in the study by Mir et al. [6] and Raineri et al. [26] experienced desaturation below 90%. The observation of no desaturation in these two studies may be because, although they included patients for emergency surgeries, BMIs were ≤35 kg/m^2^ whereas in our study the cut-off BMI was kept ≤ 40 kg/m^2^.

In concordance with the findings reported by Hua et al. [9] and Rajan et al. [28], no significant difference in hemodynamic variables was observed between the groups in our study. P-POSSUM was comparable in our study. The reason for this could be that only emergency laparotomies were included in a limited time frame in our study.

The strength of our study lies in the fact that we evaluated the efficacy of THRIVE preoxygenation in patients scheduled for emergency abdominopelvic surgeries who are generally sick with poor cardiorespiratory reserves and are at higher risk of hypoxemia during induction of anaesthesia. Our observations favoured its use as a preoxygenation tool in these patients.

The limitation of our study was we did not include pregnant patients, patients with severe cardiorespiratory diseases and morbidly obese patients with BMI ≥ 40 kg/m^2^, and thus, our results cannot guide practice in these patients. We suggest the need for future studies with larger sample sizes to investigate the efficacy of THRIVE preoxygenation in these sets of patients.

## Conclusions

Based on the observations and results of our study, we conclude that though not superior to conventional facemask, THRIVE is a safe, practicable, and efficient preoxygenation tool during rapid sequence induction of general anaesthesia for patients undergoing emergency surgeries. 
